# Fatal tumor lysis syndrome in a patient with metastatic gastric adenocarcinoma

**DOI:** 10.4322/acr.2020.225

**Published:** 2020-12-08

**Authors:** Robin Moiseff, Cameron Felty, Xiaoying Liu

**Affiliations:** 1 Dartmouth-Hitchcock Medical Center, Department of Pathology and Laboratory Medicine, Lebanon, NH, USA

**Keywords:** Stomach, Stomach Neoplasms, Tumor lysis Syndrome

## Abstract

Tumor lysis syndrome is a well-characterized and potentially deadly complication of spontaneous or treatment-related tumor destruction, and it is most commonly associated with hematologic malignancies. Our case illustrates a rare example of fatal tumor lysis syndrome in the setting of metastatic gastric adenocarcinoma treated with radiation therapy. This case highlights the critical importance of identifying patients with solid organ malignancies at risk for tumor lysis syndrome and of early recognition and treatment of this syndrome.

## INTRODUCTION

Tumor lysis syndrome is a potentially fatal complication of spontaneous or treatment-related tumor destruction, and it has been well-characterized in hematologic malignancies. The following case illustrates a unique example of fatal tumor lysis syndrome occurring a solid organ malignancy setting in a patient with metastatic gastric adenocarcinoma treated with radiation therapy. To the authors' knowledge, only 9 prior cases of tumor lysis syndrome have been reported in patients with metastatic gastric adenocarcinoma, and none of these occurred after radiation treatment alone (Table 1). This case highlights the critical importance of identifying patients with solid organ malignancies at risk for tumor lysis syndrome and of early recognition and treatment of this syndrome.

**Table 1:** Characteristics of previously reported cases of TLS in gastric adenocarcinoma from the literature.

Authors	Clinical Presentation			
	Age	Sex	Metastases	Treatment
Woo^9^	36	M	Liver, lymph nodes	None
Han^10^	38	M	Liver, lymph nodes	Cisplatin and Capecitabine
Vodopivec^8^	57	M	Liver, sternum	Oxaliplatin, Leucovorin, Floxuradine, Docetaxel
Kobayashi^11^	69	M	Liver, lymph nodes	S1 and Cisplatin
Goyal^12^	51	M	Liver, lymph nodes, L4 vertebra, adrenal gland, rectum	None (Cisplatin, Docetaxel, 5-fluorouracil 2 months prior)
Caravaca-Fontàn^13^	Unknown	Unknown	Unknown	None
Sàlmon-Gonzàlez^14^	79	M	Liver, lymph nodes	None
Lingamaneni^15^	48	M	Lymph node	5-fluorouracil, leucovorin, oxaliplatin
Chen^16^	62	M	Peritoneum, lymph nodes	None
Index case	43	M	Liver, lung, lymph nodes	X-ray Radiation Therapy

## CASE REPORT

A 43-year-old male presented to the Emergency Department (ED) with new-onset lightheadedness when standing. He had never experienced similar episodes and denied syncope. He also complained of abdominal discomfort and bloating. Past medical history was significant for untreated hypertension, morbid obesity, and obstructive sleep apnea. He described losing 11,5 kg over the preceding 6 months attributable to lifestyle changes. He was not prescribed any medications. Family history was significant for coronary artery disease, hypertension, and colorectal cancer. Complete blood count (CBC) in the ED was significant for anemia (hemoglobin 8.6 g/dL, reference value [RV]: 13.7 - 17.5 g/dL). The patient was discharged and instructed to follow up with his primary care physician to schedule an outpatient computed tomography (CT) scan.

**Figure 1:**
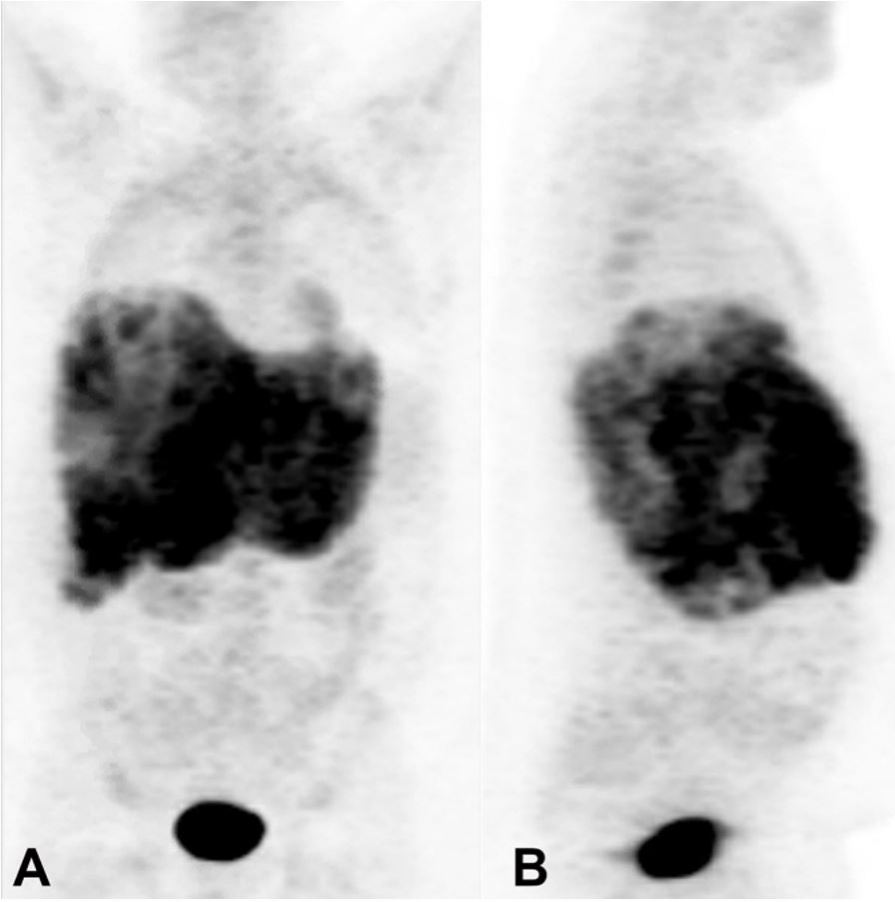
Skull base to mid-thigh PET-CT following IV injection of 18-fluoro-2-deoxyglucose (FDG) with standard uptake period **A ­** coronal and **B ­** sagittal views. Hypermetabolic thickening involving the lesser curvature of the stomach is observed, as well as numerous hypermetabolic hypodensities are throughout the liver and lymph nodes.

Two days after discharge, he presented to the outpatient clinic with continued lightheadedness and increasing exercise intolerance. Physical examination revealed pale conjunctiva, tachycardia, and guaiac positive stool. Repeat CBC revealed worsening anemia (hemoglobin 7.0 g/dL). Peripheral smear and iron studies were consistent with iron deficiency anemia. CT scan of the abdomen and positron emission tomography (PET) scan revealed thickening of the gastric cardia suspicious for malignancy along with multiple liver masses and adenopathy suspicious for metastatic disease (Figure 1).

Esophagogastroduodenoscopy (EGD) revealed a large, ulcerated mass extending from the gastric cardia into the distal esophagus. Biopsies of the mass were performed, showing poorly differentiated adenocarcinoma on histology.

Fluorescence in situ hybridization for HER2/NEU amplification was negative. The patient was subsequently treated with 5 cycles of palliative x-ray radiation therapy (XRT) to the distal esophagus/gastric cardia (total radiation dose 2000 cGy).

Upon completing cycle 5 of XRT, the patient presented to the ED. He was found to have refractory anemia (hemoglobin 6.6 g/dL), melena, and acute kidney injury with elevated serum BUN (108 mg/dL, RV: 10 - 20 mg/dL), and serum creatinine (2.03 mg/dL, RV: 0.80 - 1.50 mg/dL). During admission, the patient developed multiple electrolyte abnormalities, including hyponatremia, hyperkalemia, hyperphosphatemia, hypocalcemia, and hyperuricemia, consistent with tumor lysis syndrome (Table 2).

**Table 2:** Summary of serum abnormalities on patient’s admission following XRT.

Serum chemistries	Value	Reference value
Sodium	120 mmol/L	135-145 mmol/L
Potassium	6 mmol/L	3.5-5 mmol/L
Phosphate	12.4 mg/dl	2.5-4.5 mg/dl
Calcium	8.1 mg/dl	8.5-10.5 mg/dl
Uric acid	21.4 mg/dl	3.5-8.5 mg/dl

The patient’s kidney function continued to decline with worsening lactic acidosis, liver insufficiency, and coagulopathy with elevated INR (2.7, RV: 0.9 - 1.1) and elevated D-dimer (>20000 FEU ng/ml, RV: <200 FEU ng/ml). During this hospitalization, the patient continued to decompensate despite maximal therapy, and he subsequently expired.

## AUTOPSY FINDINGS

**Figure 2:**
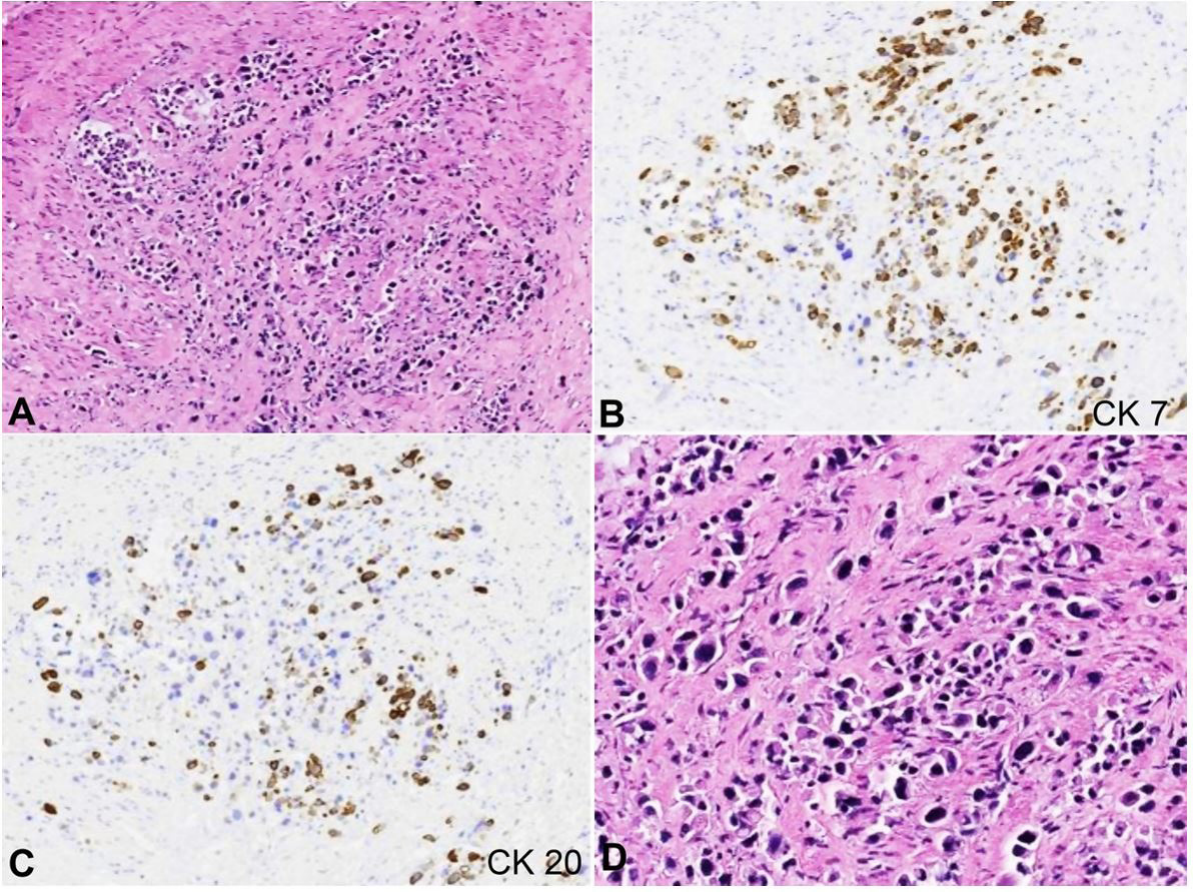
Photomicrographs of the gastric tissue showing **A** and **D ­** poorly differentiated adenocarcinoma involving the gastric cardia (H&E, 100x, 200x respectively), **B ­** strongly positive immunohistochemical staining for CK7 (100X) and **C ­** CK20 (100X).

Consent was obtained from next of kin for a limited autopsy to investigate the thorax, abdomen, and pelvis. External examination revealed anasarca and was otherwise unremarkable. The abdominal cavity contained 1800 mL of serosanguinous ascitic fluid, and the stomach contained 1100 ml of clotted blood. A 5.5 x 6.0 x 0.9 cm solid mass was present within the gastric cardia with extension into the gastroesophageal junction. The segment of the esophagus distal to the tumor exhibited mucosal hyperemia, consistent with radiation esophagitis. Sections of the gastric tumor revealed poorly differentiated adenocarcinoma with signet ring features and extensive necrosis (Figure 2).

By immunohistochemistry, the tumor was positive for cytokeratin 7 (Bond, PA0138, pre-diluted), cytokeratin 20 (Leica, NCL-L-CK20, 1:50 dilution) and also showed weak CDX-2 (Cell Marque, EPR27644, 1:100 dilution) positivity.

**Figure 3:**
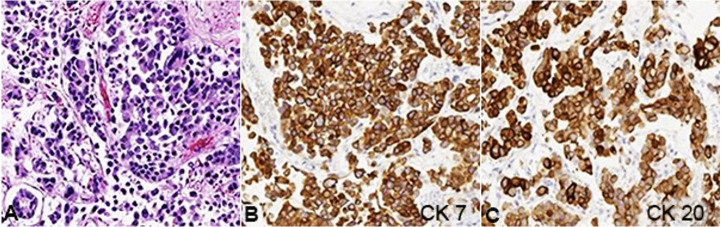
Photomicrographs of the lung tissue showing- **A ­** differentiated carcinoma (H&E, 200X), **B** strongly positive immunohistochemical staining for CK7 (200X), and **C ­** CK20 (200X).

The lungs were involved by bilateral pleural based metastatic deposits of poorly differentiated adenocarcinoma (Figure 3) measuring up to 0.9 x 0.3 cm. The tumors in the lungs were strongly positive for CK20 and CK7, and weakly positive for CDX-2 by immunohistochemistry. Staining for TTF-1 (Biogenex, MU397, 1:20 dilution) was negative, and there was an extensive lymphovascular invasion, supporting metastasis.

**Figure 4:**
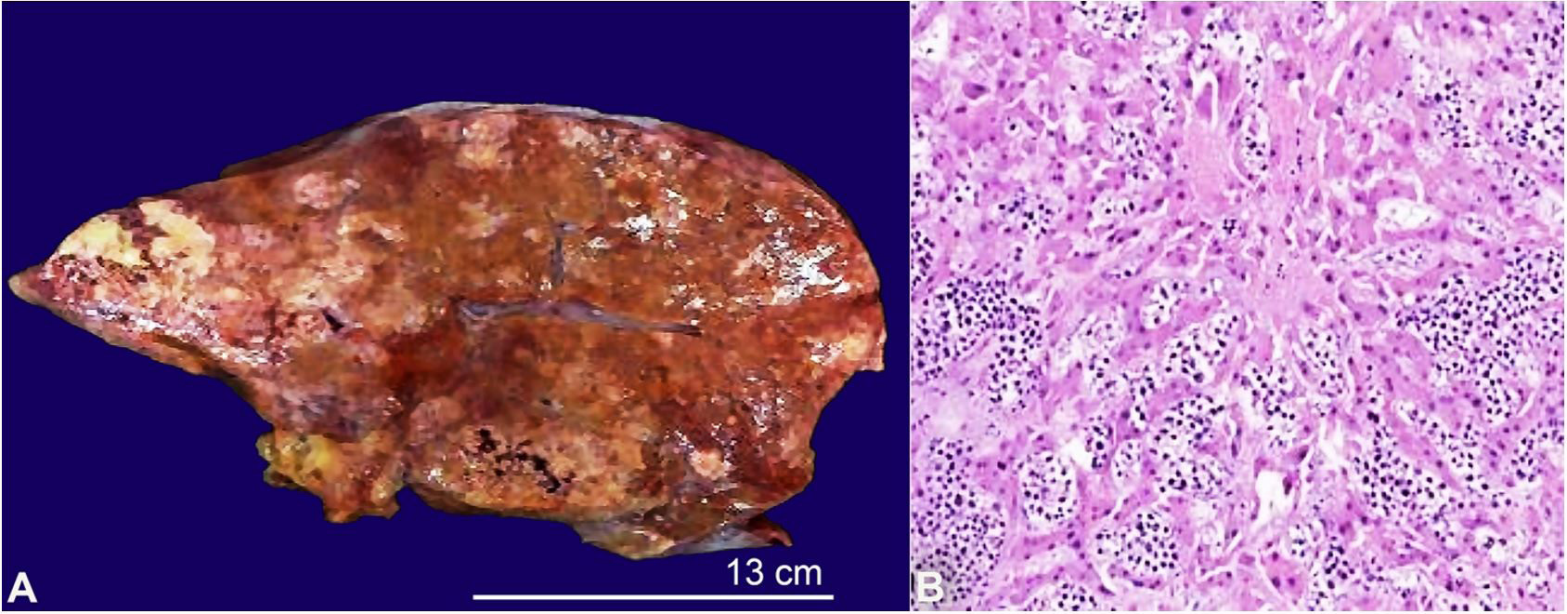
Gross autopsy photograph of the liver **A** showing diffuse involvement by rubbery tan-yellow nodular metastases with focal areas of necrosis; **B ­** Micrograph of liver showing diffuse involvement by poorly differentiated carcinoma (H&E, 100x).

The liver was grossly nodular and extensively involved by metastatic, poorly-differentiated adenocarcinoma, morphologically consistent with the gastric primary (Figure 4).

Peri-pancreatic, mesenteric, perigastric, and gastrohepatic lymph node involvement by poorly differentiated gastric adenocarcinoma was present. The kidneys were edematous with smooth subcapsular surfaces grossly. Microscopic examination of the kidneys was limited by extensive autolysis, though hypertensive vasculopathy was apparent. The heart was grossly enlarged with left ventricular hypertrophy but without significant coronary artery atherosclerosis. Histologically, the heart showed myocyte hypertrophy without evidence of acute or chronic ischemic damage.

## DISCUSSION

The laboratory and clinical definition of tumor lysis syndrome (TLS) is the development of hyperkalemia, hyperuricemia, hyperphosphatemia, and hypercalcemia with clinically significant sequelae such as acute kidney injury or cardiac dysrhythmia.^1^^-^^6^ Our patient presented with all of these metabolic disturbances by laboratory analysis and had clinical sequelae, including metabolic acidosis, renal, and hepatic failure. TLS can be a life-threatening emergency due to the severity of these end-organ effects and resulting in metabolic derangement. TLS can occur spontaneously or following treatment and is secondary to a large volume of tumor cells lysing with their components, subsequently being released into the bloodstream. It is well-described in the context of hematologic malignancies but is seen significantly less often in solid organ malignancy.^1^^-^^8^ Cases of TLS in patients with solid organ malignancies have been described as occurring sporadically, post-chemotherapy and post-radiotherapy, such as the case in our patient.^1^^-^^6^ Though cases of TLS in solid organ malignancies remain uncommon in the literature, they have occurred in many different primary sites.^1^^,^^2^^,^^7^^,^^8^ A literature review using combinations of keywords including “tumor lysis,” “tumor lysis syndrome,” “gastric adenocarcinoma,” and “gastric cancer” was performed utilizing PubMed, Scopus, Google Scholar and Web of Science. To the authors’ knowledge, there have been 9 previously reported cases of gastric adenocarcinoma with TLS (Table 1). In these cases, TLS occurred both spontaneously and secondary to chemotherapy. To our knowledge, the present case is the first reported case of TLS in gastric adenocarcinoma after only radiation therapy administration in the absence of chemotherapy.

In solid organ malignancies, risk factors for the development of TLS include high tumor burden and tumors with high proliferative/turnover rates, sometimes demonstrated by elevated lactate dehydrogenase (LDH).^1^^-^^5^ Intense initial treatment of tumor and high tumor sensitivity to treatment also increases the risk of TLS.^3^ Solid organ or hematologic malignancies with widely metastatic disease may have a larger tumor burden than the localized disease, which was the case in our patient who had a large primary gastric tumor as well as extensive nodal and hepatic metastases. Patients with dehydration, underlying kidney disease, hypotension or concurrent treatment with nephrotoxic agents are also at higher risk for TLS.^3^^,^^8^ Treatments depend on a patient’s specific electrolyte disturbance profile (calcium for hypocalcemia, potentially dialysis for severe hyperkalemia or hyperphosphatemia), but generally include supportive therapy such as aggressive fluid resuscitation, and treatment with allopurinol or rasburicase to decrease the formation and increase excretion of uric acid, respectively.^2^^,^^3^ Our patient received radiation therapy on an outpatient basis, subsequently presented to the ED with findings classic for TLS, and unfortunately had a fatal outcome.

## CONCLUSIONS

This case is unique in highlighting an unusual consequence of a metastatic gastrointestinal malignancy culminating in fatal tumor lysis syndrome after outpatient radiation therapy. This case accentuates the importance of early recognition of TLS or pre-emptively identifying at-risk patients with solid organ malignancies. This includes patients with high overall tumor burden including metastatic deposits or tumors with high proliferative rates, patients with tumors expected to be highly sensitive to treatment, and patients with underlying risk factors such as chronic kidney disease or hypovolemia. It is especially important for providers to give patients thorough instructions on potential signs of TLS, such as decreased urine output, muscle cramps, or mental changes. Providers should be cognizant that patients, such as ours, who may be undergoing treatment on an outpatient basis for solid organ malignancy, may not be receiving concurrent aggressive fluid resuscitation with treatment. As we continue to develop more efficacious treatments for aggressive malignancies, we must be acutely aware of the potential for TLS in patients with solid organ cancers.

## References

[1] McBride A, Westervelt P (2012). Recognizing and managing the expanded risk of tumor lysis syndrome in hematologic and solid malignancies. J Hematol Oncol.

[2] Baeksgaard L, Sørensen JB (2003). Acute tumor lysis syndrome in solid tumors-A case report and review of the literature. Cancer Chemother Pharmacol.

[3] Howard SC, Jones DP, Pui CH (2011). The tumor lysis syndrome. N Engl J Med.

[4] Howard SC, Trifilio S, Gregory TK (2016). Tumor lysis syndrome in the era of novel and targeted agents in patients with hematologic malignancies: a systematic review. Ann Hematol.

[5] Zafrani L, Canet E, Darmon M (2019). Understanding tumor lysis syndrome. Intensive Care Med.

[6] Cairo MS, Bishop M (2004). Tumour lysis syndrome: new therapeutic strategies and classification. Br J Haematol.

[7] Mirrakhimov AE, Ali AM, Khan M, Barbaryan A (2014). Tumor lysis syndrome in solid tumors: An up to date review of the literature. Rare Tumors.

[8] Vodopivec DM, Rubio JE, Fornoni A, Lenz O (2012). An unusual presentation of tumor lysis syndrome in a patient with advanced gastric adenocarcinoma: case report and literature review. Case Rep Med.

[9] Woo IS, Kim JS, Park MJ (2001). Spontaneous acute tumor lysis syndrome with advanced gastric cancer. J Korean Med Sci.

[10] Han HS, Park SR, Kim SY (2008). Tumor lysis syndrome after capecitabine plus cisplatin treatment in advanced gastric cancer. J Clin Oncol.

[11] Kobayashi T, Kuwai T, Yamamoto S (2012). Acute tumor lysis syndrome in the setting of advanced gastric cancer. Nihon Shokakibyo Gakkai Zasshi.

[12] Goyal H, Sawhney H, Bekara S, Singla U (2014). Spontaneous acute tumour lysis syndrome in gastric adenocarcinoma: a case report and literature review. J Gastrointest Cancer.

[13] Caravaca-Fontán F, Martínez-Sáez O, Pampa-Saico S, Olmedo ME, Gomis A, Garrido P (2017). Tumour lysis syndrome in solid tumors: clinical characteristics and prognosis. Med Clin (Barc).

[14] Salmón-González Z, Vieitez-Santiago M, Martino-González M, Hernández JL, Alonso-Gutierrez J (2019). Spontaneous tumor lysis syndrome occurring in untreated gastric adenocarcinoma. QJM.

[15] Lingamaneni P, Desai MP, Mathew M, Moturi K, Krishna R, Gupta S (2019). Tumor lysis syndrome in a patient with gastric adenocarcinoma. Am J Gastroenterol.

[16] Chen K-B, Xi W-J, Huang Y (2019). Spontaneous Tumor Lysis Syndrome in a patient with advanced gastric adenocarcinoma. Transl Cancer Res.

